# Leber hereditary optic neuropathy: bridging the translational gap

**DOI:** 10.1097/ICU.0000000000000410

**Published:** 2017-08-10

**Authors:** Neringa Jurkute, Patrick Yu-Wai-Man

**Affiliations:** a NIHR Biomedical Research Centre at Moorfields Eye Hospital and UCL Institute of Ophthalmology, London; b Wellcome Trust Centre for Mitochondrial Research, Institute of Genetic Medicine, Newcastle University; c Newcastle Eye Centre, Royal Victoria Infirmary, Newcastle upon Tyne; d Department of Clinical Neurosciences, Cambridge Centre for Brain Repair, University of Cambridge, Cambridge, UK

**Keywords:** gene therapy, Leber hereditary optic neuropathy, mitochondrial diseases, mitochondrial replacement, optical coherence tomography

## Abstract

**Purpose of review:**

Leber hereditary optic neuropathy (LHON) is the most common primary mitochondrial DNA (mtDNA) genetic disorder in the population. We address the clinical evolution of the disease, the secondary etiological factors that could contribute to visual loss, and the challenging task of developing effective treatments.

**Recent findings:**

LHON is characterized by a preclinical phase that reflects retinal ganglion cell (RGC) dysfunction before rapid visual deterioration ensues. Children can present atypically with slowly progressive visual loss or an insidious/subclinical onset that frequently results in considerable diagnostic delays. The LHON mtDNA mutation is not sufficient on its own to precipitate RGC loss and the current body of evidence supports a role for smoking and estrogen levels influencing disease conversion. Clinical trials are currently investigating the efficacy of adeno-associated viral vectors-based gene therapy approaches for patients carrying the m.11778G>A mutation. Mitochondrial replacement therapy is being developed as a reproductive option to prevent the maternal transmission of pathogenic mtDNA mutations.

**Summary:**

LHON is phenotypically more heterogeneous than previously considered and a complex interplay of genetic, environmental and hormonal factors modulates the risk of a LHON carrier losing vision. Advances in disease modelling, drug screening and genetic engineering offer promising avenues for therapeutic breakthroughs in LHON.

## INTRODUCTION

Inherited optic neuropathies are an important cause of blindness in children and young adults [1]. The advent of next-generation sequencing technology has led to a rapidly expanding list of disease causing genes and rather strikingly, all of them encode for structural or functional proteins that contribute to mitochondrial homeostasis. Leber hereditary optic neuropathy (LHON) is the classical paradigm of a mitochondrial optic neuropathy and historically, it is the first disorder for which a pathogenic point mutation was ascribed to the mitochondrial genome, which is strictly maternally inherited [2]. Much progress has been achieved since 1988 when Wallace *et al.*[3] identified the m.11778G>A mitochondrial DNA (mtDNA) mutation in the *MT-ND4* gene as the most common cause of LHON (∼60%). The m.3460G>A (*MTND1*) and m.14484T>C (*MTND6*) mutations account for ∼15% of cases each, with the remainder harbouring rarer mtDNA mutations [1].

The prevalence of LHON has been well established in Northern European populations with figures ranging from one in 30 000 to one in 50 000 [4–6]. The incidence has proven more difficult to establish in specific defined geographical regions, but an estimate of one in 1000 000 has recently been reported in the Japanese population [7^▪^].

Visual recovery in LHON is influenced by the underlying mtDNA mutation, but overall, the visual prognosis is poor and the majority of patients will remain significantly visually impaired for the rest of their lives [8]. Less than 20% of patients harbouring the m.11778G>A mutation will recover 15 letters of vision from the nadir compared with more favourable recovery rates of up to 60% for those with the less deleterious m.14484T>C mutation [9,10^▪▪^]. Treatment options remain limited and this unsatisfactory situation is further compounded by the challenging nature of genetic counselling and our incomplete understanding of the triggers for visual loss in LHON [1,2]. 

**Box 1:**
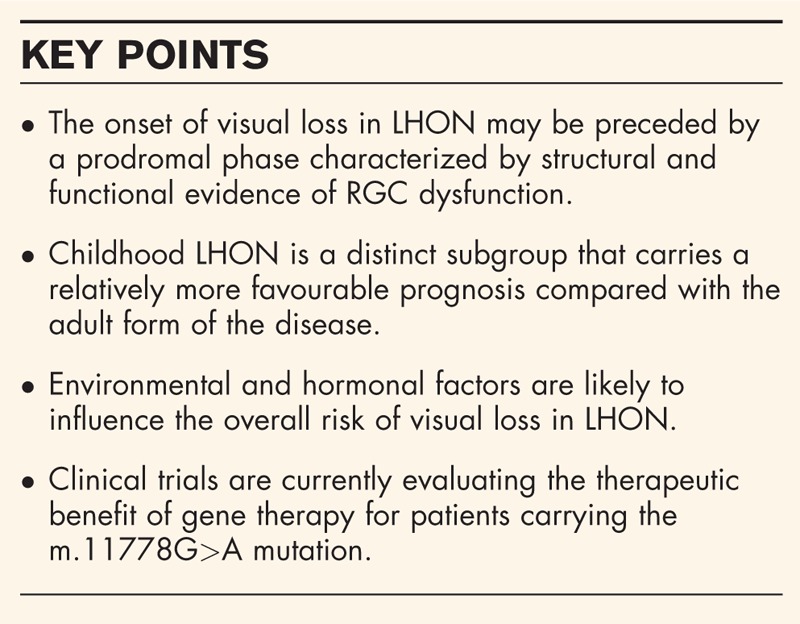
no caption available

## CLINICAL EVOLUTION

The acute stage of LHON is characterized by a rapidly evolving dense central or cecocentral scotoma. In ∼25% of cases, both eyes are affected simultaneously [1,2]. In cases with unilateral disease onset, the fellow eye is affected within 3–6 months. Although the fundus can look entirely normal, the optic disc is more commonly hyperaemic with peripapillary telangiectasias and vascular tortuosity of the central retinal vessels. There is pseudoedema secondary to swelling of the retinal nerve fibre layer (RNFL) and these morphological changes have been documented more closely with the help of optical coherence tomography (OCT) imaging. Disease conversion in LHON is heralded by swelling of the inferotemporal RNFL, which then propagates circumferentially to involve the other sectors around the optic disc [11,12]. The swelling gradually subsides and the development of optic atrophy coincides with progressive generalized RNFL thinning over the subsequent 6–12 months. The chronology and pathophysiological relevance of these retinal ganglion cell (RGC) axonal changes have been further clarified with high-resolution spectral-domain OCT imaging. It is now apparent that changes occurring at the macula, as measured by the ganglion cell and inner plexiform layer (GC-IPL) thickness, precede the wave of peripapillary RNFL swelling in the acute stage of LHON [13^▪▪^]. In patients presenting with unilateral optic nerve involvement, reduced GC-IPL thickness was noted in the fellow (unaffected) eye 6 weeks before the actual onset of visual loss and maximal thinning at the macula had already occurred within 6 months. Further work is needed to demonstrate whether GC-IPL measurements could prove a more sensitive biomarker for monitoring disease progression or detecting a therapeutic response in clinical trials.

The preclinical stage has also been defined in more detail based on the observations made in a group of six LHON carriers from a large Brazilian family carrying the m.11778A>G mutation who developed visual loss whilst being followed up as part of a prospective natural history study [14^▪▪^]. An increase in peripapillary RNFL thickness and a decrease in mean deviation of up to 5 dB on Humphrey visual field perimetry were observed 4–6 months before the patient-reported subjective visual loss. A combination of OCT and visual field changes may therefore help identify LHON carriers at high-risk for impending visual deterioration and perhaps, most likely to benefit from early intervention when the means to stop or slow RGC loss eventually becomes available.

The vascular changes in the acute stage of LHON and the transient peripapillary telangiectasias seen in asymptomatic mutation carriers are suggestive clues pointing towards a possible vasculopathy contributing to the complex pathophysiology of this disorder. Thickening of the macular and peripapillary choroidal layer has been reported in the acute stage, followed by thinning in the chronic stage [15]. The RNFL changes preceded the choroidal changes, and it remains to be determined whether the latter represents a compensatory response secondary to a state of ‘pseudo-hypoxia’. OCT angiography (OCT-A) is a promising development in ocular imaging that is being used to delineate the retinal vasculature in various pathological states, including age-related macular degeneration and diabetic retinopathy. Three case reports of OCT-A in patients with acute LHON confirm the marked vascular dilatation and tortuosity that is evident clinically [16–18]. Importantly, there is no leakage on fluorescein angiography, which differentiates LHON from other inflammatory optic neuropathies or true papilledema in the context of raised intracranial pressure. In the chronic stage of LHON, OCT-A shows extensive capillary drop out, in particular in the region of the papillomacular bundle.

## CHILDHOOD LEBER HEREDITARY OPTIC NEUROPATHY

LHON is predominantly a disease of young adults and the peak age of onset is in the second and third decades of life. Less than 10% of patients are 12 years old or younger at the time of diagnosis and previous reports have described atypical patterns of visual loss in this subgroup [19,20]. Childhood LHON seems to carry a better visual prognosis compared with the adult form of the disease. A recent meta-analysis combined a British pediatric cohort of 27 patients with 69 additional cases that were identified from a systematic review of the literature [10^▪▪^]. The inclusion criteria were onset of visual loss at 12 years old or younger and molecular confirmation of one of the three primary mtDNA mutations (m.3460G>A, m.11778G>A or m.14484T>C). The phenotype in childhood LHON could be classified into three distinct subtypes: first, classical acute, which was the most common, being seen in about two-thirds of all cases; second, slowly progressive, if visual deterioration occurred over a period exceeding 6 months and third, insidious or subclinical for those individuals who were clinically asymptomatic at the time that a diagnosis of optic atrophy or subnormal vision was made, and in whom no further decline in visual acuity occurred during subsequent follow-ups. This latter subgroup accounted for ∼20% of cases and diagnostic delays of up to 15 years were encountered. A significant proportion of patients (∼40%) achieved a final best-corrected visual acuity (BCVA) at least6/12 Snellen in at least one eye, but ∼20% of patients did poorly with a final BCVA less than 3/60 in their better seeing eye. Although childhood onset is a positive prognostic factor in LHON, a degree of caution is warranted as part of counselling given that one in five patients will remain within the visual acuity criteria for legal blindness.

From a clinical perspective, LHON can have an insidious manifestation in the paediatric population and mtDNA genetic testing should be considered when faced with a child with unexplained subnormal vision and optic disc pallor to avoid potentially prolonged diagnostic delays.

## INCOMPLETE PENETRANCE AND MALE BIAS

LHON shows variable penetrance both between families and within branches of the same family. The lifetime risk of a male carrier losing vision is ∼50% compared with ∼10% for a female LHON carrier. The secondary factors that underlie this incomplete penetrance and the strong male bias for visual loss have been the subject of intense investigation over the past 3 decades. Genetic modelling initially pointed towards a nuclear modifier gene on the X chromosome, but linkage studies have been inconclusive with different risk loci having been mapped and failure so far to identify a causative gene that segregates with affected disease status in LHON [21–23]. The current body of evidence points towards LHON being a complex disease with multifactorial genetic and environmental triggers interacting with the LHON mtDNA mutation [24]. Uncovering the nature of these modulatory factors has been difficult for several reasons, in particular, the relatively rarity of LHON for conducting adequately powered, large-scale genomic studies and the difficulty in collecting accurate environmental exposure data.

## ENVIRONMENTAL TRIGGERS

A number of retrospective epidemiological studies have provided convincing evidence that heavy smoking, and to a lesser extent excessive alcohol consumption, is a major risk factor for visual loss in LHON [25–27]. It is biologically plausible that these environmental triggers play a more prominent role for mutation carriers who experience visual loss in later life, presumably because they have a lower *a priori* nuclear genetic predisposition [28,29]. There are potential confounding factors inherent in the retrospective recall and analysis of environmental exposure, but the deleterious impact of smoking on mitochondrial function has been confirmed *in vitro* with the use of primary LHON fibroblasts established from affected and unaffected mutation carriers [30^▪^]. Cells that were cultured under the influence of cigarette smoke demonstrated a significant reduction in ATP synthesis, which is to be expected given that LHON mtDNA mutations affect critical subunits of the mitochondrial respiratory chain complexes. More prominent oxidative damage were also found in fibroblasts from affected mutation carriers implying a reduced capacity to mitigate the effect of impaired mitochondrial electron flux and the consequent toxic elevation in reactive oxygen species (ROS) levels. LHON mutation carriers should therefore be strongly advised not to smoke and to moderate their drinking habits as these are the only modifiable risk factors over which they have a personal degree of control.

## HORMONAL INFLUENCES

A longstanding hypothesis is whether the striking sex bias in LHON could arise because of hormonal differences, especially the higher levels of circulating estrogens in women. This would imply a second peak of female LHON carriers converting in the perimenopausal or menopausal period, but there is no robust data yet to suggest that this is the case. RGCs express high levels of the estrogen beta receptor and estrogen derivatives are known to exert a neuroprotective effect under conditions of heightened cellular stress [31,32]. The work of Giordano *et al.* have provided convincing experimental proof that estrogen derivatives were effective in partially rescuing the downstream consequences of LHON mtDNA mutations on mitochondrial function in a well characterized LHON cybrid model [33,34^▪^]. When treated with a specific combination of phytoestrogens, which are natural estrogen-like compounds, LHON cybrids showed improved mitochondrial respiratory chain function, lower ROS levels and a reduced sensitivity to undergo apoptosis. These promising in-vitro results will need to be pursued further and if confirmed, the next step would be to screen for estrogen derivatives that are mitochondrial protective, whilst not having any of the adverse side effects of sustained estrogen use, especially feminising effects and an increased risk of breast and gyaecological cancers if the treatment is to be given prophylactically to male and female LHON carriers, respectively.

## FROM BENCH TO BEDSIDE

LHON is a frustrating condition for patients, their families and the physicians looking after them. The stark reality is that there is currently no treatment that will reverse the catastrophic loss of RGCs that occurs rapidly in the first few weeks of disease onset [35]. The development of treatment paradigms for LHON and other mitochondrial optic neuropathies has been severely hampered by the inability to study disease mechanisms directly in RGCs and the lack of animal models that faithfully replicate the optic neuropathy and clinical manifestations.

Idebenone is a synthetic, short-chain analogue of ubiquinone, which improves the shuttling of electrons from complexes I and II directly to complex III. Idebenone is partially effective when given in the acute stage of LHON, but only a subgroup of patients experience a clinically significant visual benefit [36–38]. There is clearly the need to develop more targeted treatments that will block one or more of the key pathways that link mitochondrial dysfunction with optic nerve neurodegeneration in LHON. A combinatorial approach is likely to be necessary and the ultimate objective would be to prevent RGC loss in the first place for those LHON carriers deemed to be at high risk of disease conversion based on preexisting susceptibility factors and/or the presence of ophthalmological features presaging imminent deterioration in visual acuity.

## THERAPEUTIC DRUG SCREENING

Mitochondria exist as a highly interconnected network that extends throughout the cell and mtDNA copy number is a surrogate marker of a cell's bioenergetic demand. Significantly, mtDNA copy number was found to be significantly higher in unaffected LHON carriers compared with affected LHON carriers in a range of tissues that were tested, including peripheral blood cells, skeletal muscle biopsies and cells isolated by laser capturing postmortem specimens of retina and optic nerves [39,40]. Improved mitochondrial biogenesis could therefore be a key compensatory mechanism that protects some LHON carriers from losing vision. This hypothesis is further borne out by the emerging story with regard to tobacco toxins and estrogens, which decrease and increase mtDNA copy number, respectively, potentially accounting for their influence on disease penetrance [30^▪^,34^▪^].

A recent paper highlighted that an imbalance between nicotinamide adenine dinucleotide (NAD+) and the reduced form of NAD+ (NADH) could be the unifying link through which impaired mitochondrial oxidative phosphorylation (OXPHOS) results in RGC loss in both aged mice and a glaucoma mouse model [41^▪▪^,^42^]. Boosting the level of NAD+ by long-term dietary supplementation with the NAD+ precursor nicotinamide (vitamin B3) or gene therapy prevented RGC dysfunction and neuronal loss, providing an attractive therapeutic paradigm for glaucoma and, by extrapolation, mitochondrial optic neuropathies.

High-throughput drug screening is another possible way forward to identify molecules with a positive effect on mitochondrial biogenesis and OXPHOS, and these could then be further tested in RGCs, which are the susceptible neuronal population in LHON. Induced pluripotent stem cells can now be routinely produced by reprogramming peripheral blood cells or fibroblasts obtained from a punch skin biopsy [43]. In parallel, differentiation protocols are being refined by a number of research groups worldwide to allow the efficient generation of various retinal cells, including RGCs, providing an ideal ‘disease in a dish’ model for testing promising drug molecules in a more physiological setting [44–46].

## GENE THERAPY

Despite the complexity in unravelling the secondary modifiers that precipitate visual loss, LHON is still amenable to classical gene therapy with replacement of the missing protein product. Mitochondrial gene therapy is a challenging task because of the physical barrier imposed by having two mitochondrial membranes and the need for sustained gene expression following delivery of the gene construct into the mitochondrial matrix compartment [47]. To circumvent these technical difficulties, one ingenious option is allotopic gene expression whereby the gene construct is inserted into the nuclear genome, and the resulting protein has a mitochondrial targeting sequence, which then allows its import into the mitochondrial compartment [48]. Genetically modified adeno-associated viral vectors (AAV2) have been developed to deliver the *MT-ND4* gene construct as a therapeutic strategy to compensate for the m.11778G>A mutation [49,50]. Preclinical data have provided consistent results supporting therapeutic rescue *in vitro* and a reduction in the amount of RGCs lost in the relevant murine models tested, which was associated with an improvement in visual function. Some investigators have expressed legitimate concerns regarding the efficiency with which the imported ND4 subunit will integrate into Complex I and whether this will result in a sufficiently stable complex to promote the efficient flow of electrons along the mitochondrial respiratory chain [47,51]. Preliminary data from early-phase clinical trials support the previously established safety of AAV2-based gene therapy vectors and an encouraging visual trend for the eyes that have been treated with a single intravitreal injection [52–54]. The efficacy of this therapeutic paradigm for patients with LHON still needs to be established and three clinical trials (NCT02161380, NCT02652767 and NCT02652780, https://clinicaltrials.gov/ct2/home, accessed on 1 June 2017) are currently recruiting patients in Europe and North America to evaluate the optimal dose and the effects of treatment at different stages of the disease process.

## MITOCHONDRIAL REPLACEMENT THERAPY

LHON is a disease of young adults and reproductive choice is a topic of discussion that is frequently raised by patients and other maternally related family members during genetic counselling. Some female carriers, most of whom will be unaffected, are keen to have their own biological children, but they are understandably anxious about the future implications for their children who will be inheriting their mtDNA mutation.

Mitochondrial replacement techniques have been developed to prevent the maternal transmission of mtDNA mutations, some of which cause early-onset, severe and invariably fatal encephalomyopathies [47,55,56]. The methods being proposed are based on modification of established IVF techniques. They involve the transfer of parental nuclear material into a mitochondrial donor zygote carrying only wild-type mtDNA and the experimental data obtained so far has been encouraging with minimal carry over of mutant mtDNA [57^▪▪^,^58^^▪▪^]. In a primate model, embryonic development proceeded normally following implantation of the embryos, resulting in the births of healthy offspring [57^▪▪^,^58^^▪▪^]. This reproductive approach remains controversial as it entails germline modification with a child inheriting genetic material from a third person, albeit a limited number of 37 mitochondrial genes compared with the estimated 20 000 genes that constitute the nuclear genome. The concern also relates to the possibility that the adverse effects from this new genetic admixture might not become apparent until later in life [47,55,56]. The scientific and ethical debate as to whether mitochondrial donation should be offered to prospective mothers carrying pathogenic mtDNA mutations has accelerated further when the media released the somewhat unexpected news about the first live birth arising from mitochondrial replacement in September 2016 [59^▪▪^,^60^]. The child's mother is heteroplasmic for the m.8993T>G mtDNA mutation in *MT-ATP6*, which in addition to the syndrome of neuropathy, ataxia and retinitis pigmentosa can also cause Leigh syndrome when present at high mutant levels. Although clinically asymptomatic, she suffered from multiple miscarriages and two of her children died at the age of 8 months and 6 years old from Leigh syndrome with the m.8993T>G mutation detected at levels exceeding 95%. The newly born baby boy was reported to be healthy at 7 months age and relatively low levels of the mutation (2.4–9.2%) were detected in the tissues that were analysed. The child's neurodevelopmental progress will be carefully reviewed and fertility testing will be considered when he reaches the age of 18 years. Following an extensive public consultation exercise and a stringent scientific peer review process, the Human Fertilisation and Embryology Authority in the United Kingdom has granted a licence in March 2017 for the first clinic to be set up in Newcastle upon Tyne, United Kingdom, to provide mitochondrial donation as a reproductive option for eligible female carriers harbouring disease-causing mtDNA mutations (http://www.hfea.gov.uk/10635.html, accessed on 1 June 2017).

## CONCLUSION

The past 5 years have seen a major shift in LHON research to clinical translation and there is renewed hope that the translational gap can be bridged by bringing together scientific communities with different skill sets and fostering greater collaborations with industry, patient-led organizations and national regulatory agencies, which until recently only had a limited understanding of the unique challenges of research into rare diseases. Experimental therapies by their very nature impose a degree of calculated risks on the participants, and the onus is on the physicians and researchers involved to clearly communicate the nature of the intervention being studied, the benefits that are being contemplated, and crucially, the grey areas that will only be answered with long-term follow-up. LHON causes devastating visual loss and the urgent need to develop and validate new treatment strategies should not cloud our judgement that patient safety should always remain the abiding central factor. There will inevitably be set-backs along the way, but the major breakthroughs being achieved in the fields of ocular phenotyping, disease modelling, drug screening and genomic manipulation all point towards a building momentum that will help drive LHON research forward.

## Acknowledgements

None.

### Financial support and sponsorship

P.Y.-W.-M. is supported by a Clinician Scientist Fellowship Award (G1002570) from the Medical Research Council (MRC, UK). P.Y.-W.-M. also receives funding from Fight for Sight (UK), the UK National Institute of Health Research (NIHR) as part of the Rare Diseases Translational Research Collaboration, and the NIHR Biomedical Research Centre based at Moorfields Eye Hospital NHS Foundation Trust and UCL Institute of Ophthalmology. The views expressed are those of the author(s) and not necessarily those of the NHS, the NIHR or the Department of Health.

### Conflicts of interest

P.Y.-W.-M. holds a consultancy agreement with GenSight Biologics (Paris, France).

## REFERENCES AND RECOMMENDED READING

Papers of particular interest, published within the annual period of review, have been highlighted as:

▪ of special interest
▪▪ of outstanding interest

## References

[1] Yu-Wai-Man P Griffiths PG Hudson G Chinnery PF Inherited mitochondrial optic neuropathies. J Med Genet 2009; 46:145–158. 19001017 10.1136/jmg.2007.054270 PMC2643051

[2] Fraser JA Biousse V Newman NJ The neuro-ophthalmology of mitochondrial disease. Surv Ophthalmol 2010; 55:299–334. 20471050 10.1016/j.survophthal.2009.10.002 PMC2989385

[3] Wallace DC Singh G Lott MT Mitochondrial-DNA mutation associated with Lebers hereditary optic neuropathy. Science 1988; 242:1427–1430. 3201231 10.1126/science.3201231

[4] Man PY Griffiths PG Brown DT The epidemiology of Leber hereditary optic neuropathy in the North East of England. Am J Hum Genet 2003; 72:333–339. 12518276 10.1086/346066 PMC379226

[5] Puomila A Hamalainen P Kivioja S Epidemiology and penetrance of Leber hereditary optic neuropathy in Finland. Eur J Hum Genet 2007; 15:1079–1089. 17406640 10.1038/sj.ejhg.5201828

[6] Rosenberg T Norby S Schwartz M Prevalence and genetics of Leber hereditary optic neuropathy in the Danish population. Investig Ophthalmol Vis Sci 2016; 57:1370–1375. 27007794 10.1167/iovs.15-18306

[7▪] Ueda K Morizane Y Shiraga F Nationwide epidemiological survey of Leber hereditary optic neuropathy in Japan. J Epidemiol 2017; pii: S0917-5040(17)30074-6. doi: 10.1016/j.je.2017.02.001. [Epub ahead of print]. 10.1016/j.je.2017.02.001 PMC5565755 28392196

[8] Kirkman MA Korsten A Leonhardt M Quality of life in patients with Leber hereditary optic neuropathy. Investig Ophthalmol Vis Sci 2009; 50:3112–3115. 19255150 10.1167/iovs.08-3166

[9] Lam BL Feuer WJ Schiffman JC Trial end points and natural history in patients with G11778A Leber hereditary optic neuropathy preparation for gene therapy clinical trial. JAMA Ophthalmol 2014; 132:428–436. 24525545 10.1001/jamaophthalmol.2013.7971 PMC4266137

[10▪▪] Majander A Bowman R Poulton J Childhood-onset Leber hereditary optic neuropathy. Br J Ophthalmol 2017; pii: bjophthalmol-2016-310072. doi: 10.1136/bjophthalmol-2016-310072. [Epub ahead of print]. 10.1136/bjophthalmol-2016-310072 28314831

[11] Barboni P Savini G Valentino ML Retinal nerve fiber layer evaluation by optical coherence tomography in Leber's hereditary optic neuropathy. Ophthalmology 2005; 112:120–126. 15629831 10.1016/j.ophtha.2004.06.034

[12] Barboni P Carbonelli M Savini G Natural history of Leber's hereditary optic neuropathy: longitudinal analysis of the retinal nerve fiber layer by optical coherence tomography. Ophthalmology 2010; 117:623–627. 20031228 10.1016/j.ophtha.2009.07.026

[13▪▪] Balducci N Savini G Cascavilla ML Macular nerve fibre and ganglion cell layer changes in acute Leber's hereditary optic neuropathy. Br J Ophthalmol 2016; 100:1232–1237. 26614631 10.1136/bjophthalmol-2015-307326

[14▪▪] Hwang TJ Karanjia R Moraes-Filho MN Natural history of conversion of Leber's hereditary optic neuropathy: a prospective case series. Ophthalmology 2017; 124:843–850. 28196731 10.1016/j.ophtha.2017.01.002

[15] Borrelli E Triolo G Cascavilla ML Changes in choroidal thickness follow the RNFL changes in Leber's hereditary optic neuropathy. Sci Rep 2016; 6:37332. 27853297 10.1038/srep37332 PMC5112509

[16] De Rojas JO Rasool N Chen RWS Optical coherence tomography angiography in Leber hereditary optic neuropathy. Neurology 2016; 87:2065–2066. 27821565 10.1212/WNL.0000000000003313

[17] Gaier ED Gittinger JW Cestari DM Miller JB Peripapillary capillary dilation in Leber hereditary optic neuropathy revealed by optical coherence tomographic angiography. JAMA Ophthalmol 2016; 134:1332–1334. 27711925 10.1001/jamaophthalmol.2016.3593

[18] Takayama K Ito Y Kaneko H Optical coherence tomography angiography in Leber hereditary optic neuropathy. Acta Ophthalmol 2017; 95:e344–e345. 27778481 10.1111/aos.13244

[19] Man PYW Griffiths PG Brown DT The epidemiology of Leber hereditary optic neuropathy in the North East of England. Am J Hum Genet 2003; 72:333–339. 12518276 10.1086/346066 PMC379226

[20] Barboni P Savini G Valentino ML Leber's hereditary optic neuropathy with childhood onset. Investig Ophthalmol Vis Sci 2006; 47:5303–5309. 17122117 10.1167/iovs.06-0520

[21] Hudson G Keers S Man PYW Identification of an X-chromosomal locus and haplotype modulating the phenotype of a mitochondrial DNA disorder. Am J Hum Genet 2005; 77:1086–1091. 16380918 10.1086/498176 PMC1285165

[22] Shankar SP Fingert JH Carelli V Evidence for a novel x-linked modifier locus for Leber hereditary optic neuropathy. Ophthalmic Genet 2008; 29:17–24. 18363168 10.1080/13816810701867607

[23] Ji Y Jia X Li S Evaluation of the X-linked modifier loci for Leber hereditary optic neuropathy with the G11778A mutation in Chinese. Mol Vis 2010; 16:416–424. 20300564 PMC2838738

[24] Yu-Wai-Man P Votruba M Burte F A neurodegenerative perspective on mitochondrial optic neuropathies. Acta Neuropathol 2016; 132:789–806. 27696015 10.1007/s00401-016-1625-2 PMC5106504

[25] Tsao K Aitken PA Johns DR Smoking as an aetiological factor in a pedigree with Leber's hereditary optic neuropathy. Br J Ophthalmol 1999; 83:577–581. 10216058 10.1136/bjo.83.5.577 PMC1723036

[26] Sadun AA Carelli V Salomao SR Extensive investigation of a large Brazilian pedigree of 11778/haplogroup J Leber hereditary optic neuropathy. Am J Ophthalmol 2003; 136:231–238. 12888043 10.1016/s0002-9394(03)00099-0

[27] Kirkman MA Yu-Wai-Man P Korsten A Gene-environment interactions in Leber hereditary optic neuropathy. Brain 2009; 132:2317–2326. 19525327 10.1093/brain/awp158 PMC2732267

[28] Carelli V d’Adamo P Valentino ML Parsing the differences in affected with LHON: genetic versus environmental triggers of disease conversion. Brain 2016; 139:e17. 26657166 10.1093/brain/awv339 PMC6080496

[29] Yu-Wai-Man P Hudson G Klopstock T Chinnery PF Reply: parsing the differences in affected with LHON: genetic versus environmental triggers of disease conversion. Brain 2016; 139:e18. 26657167 10.1093/brain/awv340 PMC5839597

[30▪] Giordano L Deceglie S d’Adamo P Cigarette toxicity triggers Leber's hereditary optic neuropathy by affecting mtDNA copy number, oxidative phosphorylation and ROS detoxification pathways. Cell Death Dis 2015; 6:e2021. 26673666 10.1038/cddis.2015.364 PMC4720897

[31] Prokai-Tatrai K Xin H Nguyen V 17 Beta-estradiol eye drops protect the retinal ganglion cell layer and preserve visual function in an in vivo model of glaucoma. Mol Pharm 2013; 10:3253–3261. 23841874 10.1021/mp400313u PMC3758120

[32] Zhou XH Li F Ge J Retinal ganglion cell protection by 17-beta-estradiol in a mouse model of inherited glaucoma. Dev Neurobiol 2007; 67:603–616. 17443811 10.1002/dneu.20373

[33] Giordano C Montopoli M Perli E Oestrogens ameliorate mitochondrial dysfunction in Leber's hereditary optic neuropathy. Brain 2011; 134:220–234. 20943885 10.1093/brain/awq276 PMC3025718

[34▪] Pisano A Preziuso C Iommarini L Targeting estrogen receptor beta as preventive therapeutic strategy for Leber's hereditary optic neuropathy. Hum Mol Genet 2015; 24:6921–6931. 26410888 10.1093/hmg/ddv396

[35] Yu-Wai-Man P Votruba M Moore AT Chinnery PF Treatment strategies for inherited optic neuropathies: past, present and future. Eye 2014; 28:521–537. 24603424 10.1038/eye.2014.37 PMC4017118

[36] Klopstock T Yu-Wai-Man P Dimitriadis K A randomized placebo-controlled trial of idebenone in Leber's hereditary optic neuropathy. Brain 2011; 134:2677–2686. 21788663 10.1093/brain/awr170 PMC3170530

[37] Carelli V La Morgia C Valentino ML Idebenone treatment in Leber's hereditary optic neuropathy. Brain 2011; 134:e188. 21810891 10.1093/brain/awr180

[38] Klopstock T Metz G Yu-Wai-Man P Persistence of the treatment effect of idebenone in Leber's hereditary optic neuropathy. Brain 2013; 136:e230. 23388409 10.1093/brain/aws279 PMC3572931

[39] Giordano C Iommarini L Giordano L Efficient mitochondrial biogenesis drives incomplete penetrance in Leber's hereditary optic neuropathy. Brain 2014; 137:335–353. 24369379 10.1093/brain/awt343 PMC3914475

[40] Bianco A Bisceglia L Russo L High mitochondrial DNA copy number is a protective factor from vision loss in heteroplasmic Leber's hereditary optic neuropathy (LHON). Invest Ophthalmol Vis Sci 2017; 58:2193–2197. 28403426 10.1167/iovs.16-20389

[41▪▪] Williams PA Harder JM Foxworth NE Vitamin B-3 modulates mitochondrial vulnerability and prevents glaucoma in aged mice. Science 2017; 355:756–760. 28209901 10.1126/science.aal0092 PMC5408298

[42] Crowston J Trounce I Relief for retinal neurons under pressure. Science 2017; 355:687–688. 10.1126/science.aam7935 28209856

[43] Shi Y Inoue H Wu JC Yamanaka S Induced pluripotent stem cell technology: a decade of progress. Nat Rev Drug Discov 2017; 16:115–130. 27980341 10.1038/nrd.2016.245 PMC6416143

[44] Parfitt DA Lane A Ramsden CM Identification and correction of mechanisms underlying inherited blindness in human iPSC-derived optic cups. Cell Stem Cell 2016; 18:769–781. 27151457 10.1016/j.stem.2016.03.021 PMC4899423

[45] Maekawa Y Onishi A Matsushita K Optimized culture system to induce neurite outgrowth from retinal ganglion cells in three-dimensional retinal aggregates differentiated from mouse and human embryonic stem cells. Curr Eye Res 2016; 41:558–568. 25880804 10.3109/02713683.2015.1038359

[46] Wong RCB Lim SY Hung SSC Mitochondrial replacement in an iPSC model of Leber's hereditary optic neuropathy. Aging 2017; 9:1341–1350. 28455970 10.18632/aging.101231 PMC5425131

[47] Yu-Wai-Man P Genetic manipulation for inherited neurodegenerative diseases: myth or reality? Br J Ophthalmol 2016; 100:1322–1331. 27002113 10.1136/bjophthalmol-2015-308329 PMC5050284

[48] Manfredi G Fu J Ojaimi J Rescue of a deficiency in ATP synthesis by transfer of MTATP6, a mitochondrial DNA-encoded gene, to the nucleus. Nat Genet 2002; 30:394–399. 11925565 10.1038/ng851

[49] Guy J Qi XP Pallotti F Rescue of a mitochondrial deficiency causing Leber hereditary optic neuropathy. Ann Neurol 2002; 52:534–542. 12402249 10.1002/ana.10354

[50] Ellouze S Augustin S Bouaita A Optimized allotopic expression of the human mitochondrial ND4 prevents blindness in a rat model of mitochondrial dysfunction. Am J Hum Genet 2008; 83:373–387. 18771762 10.1016/j.ajhg.2008.08.013 PMC2556433

[51] Perales-Clemente E Fernandez-Silva P Acin-Perez R Allotopic expression of mitochondrial-encoded genes in mammals: achieved goal, undemonstrated mechanism or impossible task? Nucl Acids Res 2011; 39:225–234. 20823090 10.1093/nar/gkq769 PMC3017613

[52] Wan X Pei H Zhao MJ Efficacy and safety of rAAV2-ND4 treatment for Leber's hereditary optic neuropathy. Sci Rep 2016; 6:21587. 26892229 10.1038/srep21587 PMC4759604

[53] Yang S Ma SQ Wan X Long-term outcomes of gene therapy for the treatment of Leber's hereditary optic neuropathy. Ebiomedicine 2016; 10:258–268. 27426279 10.1016/j.ebiom.2016.07.002 PMC5006665

[54] Feuer WJ Schiffman JC Davis JL Gene therapy for Leber hereditary optic neuropathy initial results. Ophthalmology 2016; 123:558–570. 26606867 10.1016/j.ophtha.2015.10.025 PMC5287094

[55] Chinnery PF Zeviani M Mitochondrial matchmaking. N Engl J Med 2016; 375:1894–1896. 27959648 10.1056/NEJMcibr1608715

[56] Morrow EH Reinhardt K Wolff JN Dowling DK Risks inherent to mitochondrial replacement. EMBO Rep 2015; 16:541–544. 25807984 10.15252/embr.201439110 PMC4428046

[57▪▪] Hyslop LA Blakeley P Craven L Towards clinical application of pronuclear transfer to prevent mitochondrial DNA disease. Nature 2016; 534:383–386. 27281217 10.1038/nature18303 PMC5131843

[58▪▪] Kang EJ Wu J Gutierrez NM Mitochondrial replacement in human oocytes carrying pathogenic mitochondrial DNA mutations. Nature 2016; 540:270–275. 27919073 10.1038/nature20592

[59▪▪] Zhang J Liu H Luo S Live birth derived from oocyte spindle transfer to prevent mitochondrial disease. Reprod Biomed Online 2017; 34:361–368. 28385334 10.1016/j.rbmo.2017.01.013

[60] Alikani M Fauser BC Garcia-Valesco JA First birth following spindle transfer for mitochondrial replacement therapy: hope and trepidation. Reprod Biomed Online 2017; 34:333–336. 28385333 10.1016/j.rbmo.2017.02.004

